# Recent advances in regulating the proliferation or maturation of human-induced pluripotent stem cell-derived cardiomyocytes

**DOI:** 10.1186/s13287-023-03470-w

**Published:** 2023-08-30

**Authors:** Hao Yang, Yuan Yang, Fedir N. Kiskin, Mengcheng Shen, Joe Z. Zhang

**Affiliations:** 1 Institute of Neurological and Psychiatric Disorders, Shenzhen Bay Laboratory, Shenzhen, 518132 China; 2 Stanford Cardiovascular Institute, Stanford University School of Medicine, Stanford, CA USA

**Keywords:** Human-induced pluripotent stem cells, Cardiac differentiation, Cardiomyocytes, Proliferation, Maturation

## Abstract

In the last decade, human-induced pluripotent stem cell-derived cardiomyocyte (hiPSC-CM)-based cell therapy has drawn broad attention as a potential therapy for treating injured hearts. However, mass production of hiPSC-CMs remains challenging, limiting their translational potential in regenerative medicine. Therefore, multiple strategies including cell cycle regulators, small molecules, co-culture systems, and epigenetic modifiers have been used to improve the proliferation of hiPSC-CMs. On the other hand, the immaturity of these proliferative hiPSC-CMs could lead to lethal arrhythmias due to their limited ability to functionally couple with resident cardiomyocytes. To achieve functional maturity, numerous methods such as prolonged culture, biochemical or biophysical stimulation, in vivo transplantation, and 3D culture approaches have been employed. In this review, we summarize recent approaches used to promote hiPSC-CM proliferation, and thoroughly review recent advances in promoting hiPSC-CM maturation, which will serve as the foundation for large-scale production of mature hiPSC-CMs for future clinical applications.

## Introduction

**Fig. 1:**
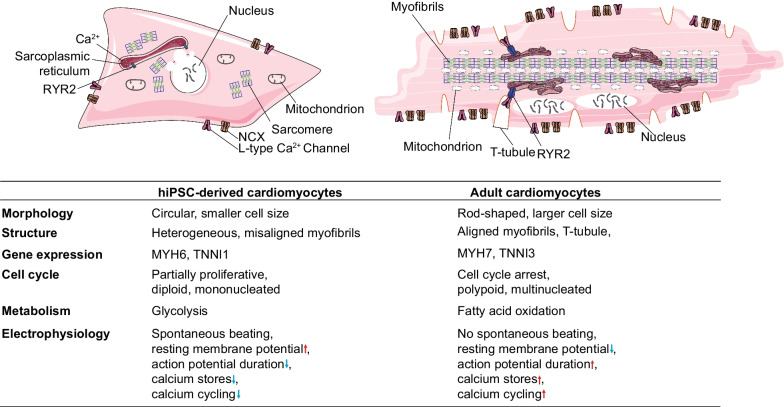
Comparison of hiPSC-CMs with adult CMs in terms of morphology, structure, gene expression, cell cycle, metabolism, and electrophysiology. NCX, sodium–calcium exchanger; RYR2, ryanodine receptor 2; MYH6, myosin heavy chain 6; MYH7, myosin heavy chain 7; TNNI1, troponin I1, slow skeletal type; TNNI3, troponin I3, cardiac type. Red arrows indicate upregulation, and blue arrows indicate downregulation. The figure was drawn by the authors in Adobe Illustrator

The ability of human-induced pluripotent stem cells (hiPSCs) to differentiate into nearly all cell types in the heart has revolutionized the field of cardiovascular regenerative medicine [1, 2]. To date, multiple protocols have been developed to differentiate hiPSCs into cardiomyocytes (hiPSC-CMs) with high purity [3–6]. However, generating adequate quantities of autologous hiPSC-CMs for high-throughput drug screening and cell therapy remains a challenge as the differentiation process is labor-intensive, time-consuming, and costly. Meanwhile, hiPSC-CMs will lose their proliferative capacity with long-term culture, restricting further expansion [7, 8]. Moreover, the low survival rate of transplanted hiPSC-CMs in the host heart suggests that it should be beneficial to preserve a certain degree of proliferative capacity of hiPSC-CMs to enhance engraftment [9]. Thus, developing effective strategies to promote hiPSC-CM proliferation is of great importance to mitigate the unmet research and clinical needs. On the other hand, current methods to differentiate hiPSC-CMs from hiPSCs mainly mimic the process of embryonic development, leading to the generation of fetal-like immature CMs in terms of their morphology, structure, metabolism, and electrophysiology when compared with adult CMs (Fig. 1). These immature hiPSC-CMs could be the source of lethal cardiac arrhythmias when transplanted into large animal hearts such as non-human primates, which largely precludes their applications in human myocardial injury therapies [10]. Finally, immature hiPSC-CMs cannot fully recapitulate the disease phenotypes and pharmacological responses of adult primary CMs, further limiting their use in pre-clinical applications [11, 12]. Therefore, efforts to develop novel methods to promote the proliferation capacity of hiPSC-CMs at an early stage and improve their maturation before use are crucial to circumventing the current limitations in the regenerative medicine research field.

In this review, we systematically detail the current progress in developing methods to promote hiPSC-CM proliferation, summarize recent advances in improving hiPSC-CM maturation, and conclude by outlining future prospects for the field.

## Regulators of hiPSC-CM proliferation and maturation

### Cell cycle regulators

In mammals, embryonic, fetal, and early postnatal CMs are proliferative; however, they quickly lose their proliferative potential over a short period of time after birth by exiting the cell cycle and shifting into the maturation program to adapt to the challenging environment and increasing workload [13]. Therefore, CMs start to lose their proliferative capacity when they become more mature. hiPSC-CMs cultured in vitro mirror the in vivo maturation process of their primary counterparts. The expression levels of cyclins and cyclin-dependent kinases (CDKs) decrease during hiPSC-CM differentiation, and the proliferation rate of hiPSC-CMs gradually decreases over prolonged periods in culture [7, 8, 14]. Ji and colleagues found that the compound CY116, which inhibits the activity of Aurora kinase which controls cell division, can promote the maturation of hiPSC-CMs [15]. A recent study also showed that the sarcomere assembly inhibited mitosis and promoted polyploidization by p53 activation and cyclin B1 (CCNB1) repression, indicating that cell cycle maintenance conflicts with myofibril formation [16]. Therefore, re-introducing hiPSC-CMs into the cell cycle might be a feasible strategy to enhance proliferation. Landmark studies by Zhang’s group showed that cyclin D2 (CCND2) overexpression activated cell cycle progression in hiPSC-CMs and that transplanting these CCND2-overexpressing hiPSC-CMs into mouse or swine hearts with myocardial infarction significantly enhanced myocardial repair [17, 18]. More importantly, although all swine hearts showed severe arrhythmias and ST segment elevations attributable to the acute ischemia/reperfusion injury, no spontaneous arrhythmia was observed during the four-week follow-up period by continuous electrocardiogram (ECG) recordings [17]. A possible explanation is that a smaller number of CCND2-overexpressing hiPSC-CMs (10^7^ *vs* 10^8^ compared to other studies) are sufficient for the cell therapy, demonstrating the advantage of preserving a certain degree of proliferative capacity of hiPSC-CMs in cardiac repair [17, 19, 20]. Further pre-clinical studies are needed to confirm the therapeutic effect of CCND2-overexpressing hiPSC-CMs, especially long-term assessment of tumorigenicity and arrhythmogenic potential of proliferating hiPSC-CMs, which are essential for the application of cardiac cell therapy in human. In addition, despite being well studied in adult CMs, the functions of other cell cycle regulators, such as cyclin A2 (CCNA2), cyclin G1 (CCNG1), cyclin-dependent kinase 1, and 4 (CDK1 and CDK4) in regulating hiPSC-CM proliferation remain unknown [21–23]. Recent work by Bergmann’s group provided an optimal tool for enabling further validation of the cell cycle regulators of hiPSC-CM proliferation [24]. Briefly, they generated a dual reporter (mCherry-hCdt1 and mVenus-hGem) driven by the cardiac troponin T2 (TNNT2) promotor to dynamically monitor the cell cycle in hiPSC-CMs and found that G2 arrest might be responsible for the cell cycle arrest [24]. Further validation of the platform showed that the alpha-adrenergic agonist clonidine can promote hiPSC-CM proliferation [24]. Interestingly, although clonidine can also induce cell cycle activity in both in vivo and in vitro neonatal mouse cardiomyocytes, no increase in cytokinesis was observed, indicating that clonidine is not able to promote the proliferation of CMs undergoing maturation [24]. Therefore, additional compounds aimed at promoting hiPSC-CM proliferation need to be screened and validated.

### Small molecules

Decades of intensive studies have revealed that CM proliferation in vivo is regulated by multiple signaling pathways, which provide several clues for further screening pro-proliferative compounds to induce the proliferation of hiPSC-CMs (reviewed previously [25]). Due to the advances in high-throughput screening techniques, an increasing number of molecules/factors that induce hiPSC-CM proliferation have been identified. Using a high-density micro-bioreactor array, Titmarsh et al*.* screened agonists targeting Wnt, Hedgehog, IGF, and FGF pathways and found that a Wnt activator, CHIR99021 (CHIR), had the greatest influence on promoting hiPSC-CM proliferation, which was also confirmed in engineered cardiac micro-tissues [26, 27]. However, one of the disadvantages of CHIR-treated hiPSC-CMs is the reduced contractile force indicated by the lower expression of mature or contractile-related genes such as ryanodine receptor 2 (RYR2), myosin light chain 2 (MYL2), and troponin I3 (TNNI3) [28]. Intriguingly, the authors also found that engineered heart tissues (EHTs, which can promote hiPSC-CM maturation) from CHIR-treated hiPSC-CMs had slightly enhanced functional properties compared with EHTs from hiPSC-CMs without treatment, highlighting the feasibility of expanding and subsequently stimulating the maturation of hiPSC-CMs [28]. Furthermore, Mills and colleagues employed a 3D cardiac organoid platform for compound screening and identified three compound hits targeting p38α/β, purinergic receptor P2RX7, and the transforming growth factor β receptor (TGF-βR)/bone morphogenetic protein receptor (BMPR) signaling pathway, respectively, which can promote hiPSC-CM proliferation without compromising the contractile force [29]. Deeper investigation elucidated that the mevalonate pathway was the core component of the proliferation signature under these pro-proliferative stimuli, suggesting that controlling metabolism has great potential for promoting hiPSC-CM proliferation [29]. In the future, more investigations are needed to determine how intermediate metabolites regulate hiPSC-CM proliferation.

The Hippo signaling pathway, another key pathway regulating CM proliferation, is highly conserved in mammals and modulates embryonic heart development and organ size [30]. Studies have shown that genetic deletion or knockdown of key components in the pathway, such as mammalian ste20-like kinases (MST), large tumor suppressor homolog (LATS), and Salvador, resulted in cell cycle re-entry of mammalian CMs, as did the activation of YAP [31, 32]. Similarly, several small molecules such as TT-10 stimulated hiPSC-CM proliferation by activating YAP [29, 33]. However, it is important to note that not all drugs targeting the Hippo signaling pathway can promote proliferation. For instance, MST inhibitors such as compound 51 and XMU-XP-1 failed to activate hiPSC-CM proliferation due to off-target effects on many pivotal cell cycle genes [29]. Recently, Kastan and colleagues have chemically modified a LATS inhibitor, TRULI [34]. Its derivative, TDI-011536, showed improved potency and physical-chemical properties and initiated the proliferation of CMs in adult mice following cardiac cryolesions, suggesting that drug modification might provide more possibilities for improving the effects of pro-proliferative compounds [34]. Taken together, despite the discovery of an increasing number of pro-proliferative compounds [35, 36], our understanding of the mechanisms behind their pro-proliferative effects is still in its infancy. In addition, it is crucial to investigate the function of these treated hiPSC-CMs to validate their potential use in disease modeling and drug screening by confirming their ability to recapitulate the disease phenotype or drug response.

While embryonic and fetal heart development studies provide the basis for developing methods to promote hiPSC-CM proliferation, studies of postnatal heart development shed light on hiPSC-CM maturation. Thyroid hormone (THs) levels increase rapidly after birth and are essential for heart developmental processes such as the regulation of titin isoform switching from the fetal N2BA isoform to the adult N2B isoform [37]. Treatment of hiPSC-CMs with triiodothyronine (T3) significantly increased cell size, sarcomere length, contractile force, and improved calcium handling [38, 39]. Glucocorticoids, which rise before birth and remain elevated postnatally, have also been reported to promote heart maturation [40]. A combination of T3 and glucocorticoids significantly promoted T-tubule formation, which further contributed to the enhanced excitation–contraction coupling and synchronized intracellular calcium release in hiPSC-CMs, highlighting the importance of these hormones for improving hiPSC-CM maturation [41]. Other hormones such as insulin-like growth factor 1 (IGF1) can promote physiological CM hypertrophy and in combination with neuregulin 1β (NRG1) could further boost the size and contractility of human embryonic stem cell (hESC)-derived cardiac tissue, suggesting a potential role of IGF1 in regulating cardiac tissue maturation [42, 43]. Moreover, other studies found that IGF1 can also promote hESC-CM proliferation by activating the PI3K/AKT pathway, whilst blocking this pathway can reduce the effect of IGF1 and promote maturation [44, 45]. These studies indicate the multifaceted roles of IGF in regulating CM maturation and proliferation.

In addition to hormones, many small molecules have also been used to promote hiPSC-CM maturation. For example, an estrogen-related receptor gamma (ERRγ) agonist enhanced hiPSC-CM maturation, as indicated by a switch of the sarcomeric protein troponin I (TNNI) isoform from *TNNI1* to *TNNI3* and the alteration of main metabolic substrates [46]. Sakamoto and colleagues then showed that ERRγ can interact with cardiogenic factor GATA4 to orchestrate hiPSC-CM maturation [47]. A recent study further showed that in vivo both ERR α and γ are not only important for cardiac maturation but also for ventricular identity, demonstrating the key role of ERR in cardiac development [48]. Furthermore, torin1, an inhibitor of mTOR signaling, was also shown to increase TNNI3 expression, contraction, and the maximum oxygen consumption rate of hiPSC-CMs, all of which are indicators of CM maturation [49].

One of the hallmarks of CM maturation is the metabolic transition from glycolysis to fatty acid oxidation [50]. Hu and colleagues demonstrated that in a standard culture medium containing glucose, the hypoxia inducible factor 1 subunit alpha (HIF1α)-lactate dehydrogenase A axis prevented the metabolic maturation of hiPSC-CMs, while a fatty acid-rich medium without glucose promoted metabolic and functional maturation of hiPSC-CMs by shifting energy production from aerobic glycolysis to oxidative phosphorylation [51]. Subsequent studies optimized the fatty acid component of the maturation medium and found that these metabolically matured hiPSC-CMs showed improved phenotypic manifestation of several diseases such as long QT syndrome and dilated cardiomyopathy [52–54]. In addition, the peroxisome proliferator activated receptor (PPAR) signaling pathway was shown to activate fatty acid oxidation and improve in vivo CM maturation [50]. Xu and colleagues confirmed that HIF1α inhibition in combination with PPARα activation improved mitochondrial maturation and increased Ca^2+^ transient kinetics, contraction, and relaxation velocities in hiPSC-CMs [55]. Interestingly, a recent study compared the effects of different PPAR isoforms on hiPSC-CMs and found that neither PPARα or PPARβ alone promoted hiPSC-CM maturation, whereas PPARδ activation per se enhanced oxidative metabolism, contractile force, and calcium handling, although no change in conduction velocity by multielectrode array was observed. Taken together, these findings suggest that PPAR has isoform-specific effects on hiPSC-CM maturation [56].

In summary, an increasing number of small molecules have been identified which significantly augment hiPSC-CM proliferation or maturation. Future studies need to establish how cells sense these drugs, hormones, and metabolites and which cytoplasmic and nuclear targets regulate the maturation state of hiPSC-CMs.

### Long-term culture

In humans, it takes years for CMs to become fully mature, suggesting that prolonged culture could stimulate hiPSC-CM maturation [57]. In early studies, when CMs derived from embryoid bodies were cultured for 60 days, cardiomyocytes with a larger cell size, well-organized sarcomeres, and reduced cell cycle activities were noted [58]. Further prolonged culture showed additional improvements such as enhanced calcium handling properties, action potential amplitudes, and upstroke velocity [7, 8]. While hiPSC-CMs cultured for 180 days displayed mature sarcomere Z-lines, H-zone, A- and I-bands, M-lines could only be detected in a minority of hiPSC-CMs after 1 year of culture [8]. However, even after long-term culture, T-tubules, invaginations of the plasma membrane which facilitate excitation–contraction coupling, were still not observed in hiPSC-CMs [59]. To understand how long-term culture could promote hiPSC-CM maturation, Wu and colleagues built a transcriptomic landscape of hiPSC-CMs at different time points and found that hiPSC-CMs displayed higher rates of oxidative phosphorylation and β-oxidation at Day 200 compared to Day 30 of differentiation [60]. In-depth pathway analysis revealed that protein kinase A (PKA)-proteasome and heat shock protein 90 (HSP90)-dependent signaling pathways were responsible for improving mitochondrial function [60]. In summary, long-term culture can lead to a certain degree of hiPSC-CM maturation, but it is time-consuming and not all hiPSC-CMs that have been cultured long-term reach the mature state [61].

### Epigenetic regulation

Epigenetic regulation such as DNA methylation, histone modification, chromatin remodeling, and non-coding RNAs plays a critical role in the development and regeneration of the heart [62, 63]. In particular, robust progress has been made in the past few years to show the powerful influence of microRNAs (miRNAs) on hiPSC-CM proliferation or maturation [64–66]. Using thymidine 5-ethynyl-20-deoxyuridine (EdU) staining in combination with cytoplasmic carboxyfluorescein succinimidyl ester (CFSE) fluorescence to assess DNA synthesis and cytokinesis, respectively, 96 miRNAs which increase the proliferation of hiPSC-CMs were identified [65]. Further studies showed that overexpression of miR-199a or miR-302d activated cell cycle re-entry and increased the number of Ki67-positive hiPSC-CMs [64, 66]. It is worth noting that miR-199a and miR-302a enhanced hiPSC-CM proliferation by targeting different genes in the Hippo pathway, demonstrating the redundancy of miRNA-mediated regulation of Hippo signaling in the context of hiPSC-CM proliferation. By contrast, a study found that 23 miRNAs were highly expressed in human embryonic stem cell-derived cardiomyocytes (hESC-CMs), fetal and adult CMs, but not in hESCs [67]. Among these miRNAs, further studies identified that overexpression of miR-1, let 7i, and miR-452 or knockout of miR-122 and miR-200a increased cell size, elevated fatty acid usage, and enhanced contractile force in hiPSC-CMs or hESC-CMs [67–69]. Further work is required to decipher how different miRNAs affect the different states of hiPSC-CMs and thereby strengthen our understanding of the regulation of proliferation and maturation.

In addition, we are still in the early days of knowing how other epigenetic regulators contribute to hiPSC-CM proliferation and maturation. Through in-depth profiling of the heart at different developmental stages, Olson’s and Hein’s groups demonstrated that DNA methylation and histone modification regulated gene expression during heart regeneration and development [70, 71]. Treatment of hESC-CMs with valproic acid, which induces trimethylation of lysine 4 on histone H3 (H3K4me3), led to a significant increase in CM size and stimulated the expression of ion channel and calcium handling genes such as sodium voltage-gated channel alpha subunit 5 (SCN5A), potassium inwardly rectifying channel subfamily J member 2 (KCNJ2), and ryanodine receptor 2 (RYR2) [72], demonstrating the role of histone modification in hESC-CM maturation. Moreover, in recent years, epi-transcriptomic modification has garnered significant attention due to the discovery of reversible RNA methylation [48, 73, 74]. Of particular interest is RNA N6-methyladenosine (m6A) modification, which is the most abundant modification in eukaryotic mRNA and involves writers, erasers, and readers, which add, remove, and recognize m6A modifications, respectively [73]. Its continuous and dynamic regulation has a profound impact on various biological processes by governing mRNA stability, maturation, splicing, transport, and translation [75, 76]. Recent research by Yang and colleagues profiled the m6A modification of neonatal hearts and found that m6A levels dramatically decreased in Day 7 hearts compared to Day 0 hearts [77]. Further studies revealed that Abraxas 2 (ABRO1) was upstream of METTL3 (writer), which restricts METTL3 activity in postnatal CMs, whilst the METTL3—miR-17-3p axis promoted proliferation [78, 79]. However, several studies also showed that METTL3 inhibited the proliferation capacity of CMs and that blocking METTL3 promoted heart regeneration [80, 81]. Therefore, further research is needed to fully understand the effect of METTL3 on the proliferation of CMs. Nevertheless, these studies confirmed that m6A modification plays a vital role in CM proliferation and maturation in vivo. In terms of hiPSC-CMs, Cai’s group recently identified that ALKBH5 (eraser) played a vital role not only in cardiac lineage commitment of hESCs but also in the proliferation of hiPSC-CMs [82, 83]. A more comprehensive characterization of targets and mechanisms is needed to provide more avenues to promote hiPSC-CM maturation or proliferation.

### Co-culture

The heart is composed of multiple cell types in addition to CMs. Non-myocytes such as cardiac fibroblasts (CFs), epicardial cells (EPIs), smooth muscle cells (SMCs), and endothelial cells (ECs) play a pivotal role in heart development [84, 85]. Among them, EPIs are one of the first cell types to emerge in the vertebrate heart during embryonic development and give rise to epicardial-derived cells such as SMCs and CFs during cardiogenesis [86]. To identify the potential effects of non-myocytes on hiPSC-CMs, Floy and colleagues co-cultured hiPSC-derived cardiac progenitor cells (CPCs) with EPIs for two weeks and this direct cellular cross-talk promoted CM proliferation [87]. In contrast, indirect co-culture or epicardial conditioned medium did not induce hiPSC-CM proliferation, suggesting that direct cell–cell contact and interaction is essential for EPI-mediated pro-proliferative effects on hiPSC-CMs [87]. However, Tan et al. suggested that IGF2 secreted by pre-EPIs might exert a pro-proliferative effect on hiPSC-CMs in the co-culture system [88]. The discrepancies between the two studies might be explained by the different developmental stages of cardiac cells used in the co-culture system (Floy et al. used EPI + CPC, whereas Tan et al. cultured pre-EPIs + CM) and the heterogeneity of EPIs derived from different differentiation protocols [87, 88]. Beyond that, some recent in vivo studies highlighted the significant role of cytokines derived from EPIs in heart regeneration, suggesting that the EPI-mediated paracrine effects might be a main driving force to activate CM proliferation [89, 90].

Cardiac fibroblasts are another major cell type in the heart. Ieda and colleagues deciphered that CFs have a stage-specific function in mice: embryonic CFs promote CM proliferation by secreting extracellular matrix factors, while in adult hearts, CFs induce CM hypertrophy and sarcomere organization by secreting growth cytokines, demonstrating the paracrine effects of CFs on CM structure and morphology [91]. Therefore, in-depth investigations to reveal physiological differences between embryonic and adult CFs may provide more avenues to scale up hiPSC-CM production or enhance their maturity. A recent study showed that follistatin like 1 (FSTL1), a key glycoprotein maintaining cardiac growth and development, was secreted by CFs under hypoxic conditions and promoted the proliferation of hiPSC-CMs co-cultured with CFs [92]. Apart from CFs, ECs are also drawing researchers’ attention. Co-culturing CPCs with ECs for two weeks promoted hiPSC-CM maturation properties, including cell size, gene expression, and T-tubule like structure; however, the underlying mechanisms remain unknown [93]. Several in vivo studies have suggested that paracrine signals from ECs, such as nitric oxide, prostaglandin (PG) E2, and parathyroid hormone-related peptide, are essential for the post-natal development of the heart, suggesting that paracrine factors might be the main driving force for regulating CM function in the co-culture system [94].

Taken together, these studies indicate that paracrine factors secreted by non-myocytes in co-culture systems play a key role in promoting hiPSC-CM proliferation or maturation. Further studies are required to identify which signals from non-myocytes are involved in driving hiPSC-CM maturation or proliferation, which in turn would benefit the maturation or scaling up of hiPSC-CMs by directly using these paracrine factors to exclude the contamination of other cells. Whether other cell types such as immune cells also regulate CM function remains unclear and warrants further studies.

### Substrate stiffness, physiological pattern, and conductivity

Extracellular matrices mediate tissue stiffness in the heart, leading to an elastic modulus of 10 ~ 25 kPa, while the plastic plates used for cell culture in vitro are much stiffer (~ 100 MPa) [95]. Ribeiro and colleagues cultured single hiPSC-CMs on a polyacrylamide hydrogel with a physiological stiffness (10 kPa) and observed improved sarcomere assembly, enhanced contractility and calcium handling, and well-organized mitochondria in single hiPSC-CMs [96]. Using a similar strategy, Strimaityte and colleagues recently developed an improved method in which they engineered hiPSC-CMs into cardiac microfibers to enable end-to-end connection, thereby further improving the maturation of the cardiac microfibers indicated by T-tubule and gap junction formation [97]. To better mimic the in vivo micro-environment, Afzal and colleagues re-analyzed several adult human heart transcriptomic datasets and identified several candidate genes which were highly expressed in adult CMs compared to hiPSC-CMs [98]. Based on that, they then generated a substrate called “cardiac mimetic matrix (CMM)” by conjugating arginylglycylaspartic acid (RGD), GFPGER (a commercial collagen mimetic), and nephronectin in equal amounts with hydrogel patterns into nano-architecture arrays [98]. Further maturation of hiPSC-CMs on CMM for two weeks resulted in metabolic and functional maturation, indicated by systemic transcriptomic maturation, higher oxidative phosphorylation and fatty acid oxidation, enhanced redox handling capability, and improved calcium handling [98]. Polydimethylsiloxane (PDMS) is another material used to generate soft cell substrate conditions and has been shown to trigger structural and electrophysiological maturation [99, 100]. Dhahri and colleagues recently modified this method to manufacture mature hiPSC-CMs at a large scale [101]. The authors further explored the ability of the matured hiPSC-CMs to promote cardiac repair in vivo and observed improved sarcomeric structure and alignment, enhanced host-graft electromechanical integration and electrophysiological function, and a larger increment in contractile recovery [101]. A limitation of this study is that guinea pigs are not the ideal model to assess arrhythmogenicity because of their fast heart rate. Further evaluations using non-human primates are needed. Taken together, these studies indicate that manipulating the substrate stiffness can improve hiPSC-CM maturation. More mechanistic studies will provide insights into how substrate stiffness affects CM function.

In vivo, adult CMs are elongated and rod-shaped (length: width aspect ratio of 7:1), which facilitates myofibril alignment and contractility, whereas in vitro hiPSC-CMs are circular or triangular and require physical cues to adopt a rod shape [102]. Patterning hiPSC-CMs on rectangular micro-patterns or uniaxially aligned ridges and grooves improves sarcomere organization and contractile and electrophysiological functions compared to CMs cultured in a 2D format [103–105]. Ribeiro and colleagues then cultured single hiPSC-CMs with aspect ratios (length: width) of 3:1–7:1 and found that hiPSC-CMs with a 7:1 aspect ratio displayed the highest sarcomere activity and improved myofibril alignment, demonstrating that physiological pattern formation leads to mature myofibril organization and cell function [96].

In addition to stiffness and pattern regulation, hiPSC-CM maturation is also affected by the electrical properties of the cell culture chamber/cell substrate. Dvir and colleagues incorporated gold nanowires within alginate scaffolds to improve electrical communication between adjacent cardiac cells and found that the expression levels of genes involved in muscle contraction and electrical coupling in hiPSC-CMs were upregulated [106]. The limitations of this material are that it is expensive and that cracks can easily form within the thin electroconductive metal layers, which can reduce reliability [107]. To overcome these problems, Liu and colleagues developed a platform called “AgNWs-E-PDMS”, in which they integrated a nano-textured poly-dimethylsiloxane cantilever with an embedded silver nanowire [108]. hiPSC-CMs cultured on this platform showed more synchronized beating and calcium transient signals [108]. Moreover, improving the electrical signal transduction capacity of hiPSC-CMs can also promote maturation [109]. To this end, Sottas and colleagues have reported that overexpression of connexin-43 (GJA1/CX43), which is the predominant gap junction protein in ventricular myocytes, significantly enhanced gap junction formation and electrical coupling between hiPSC-CMs [110]. However, the intercalated disc, an important structure between adjacent adult primary CMs which contributes to electrical conduction, was hardly observed in hiPSC-CMs, even after stimulating their maturation. Therefore, further investigation to understand how this special and complex structure is assembled in vivo may provide more clues to promote the formation of intercalated discs in hiPSC-CMs.

Taken together, these studies demonstrate that the characteristics of the biomaterials used to culture hiPSC-CMs play a vital role in promoting the maturation of hiPSC-CMs.

### In vivo maturation

The in vivo environment provides the elements required for guiding CM maturation, indicating that exposure of hiPSC-CMs to the same in vivo micro-environment might confer similar maturation effects. Indeed, researchers have found that hPSC-CM maturation was enhanced after transplantation into hearts for cardiac repair [20, 111, 112]. However, Kadota and colleagues found that only partially matured hiPSC-CM myofibrils developed three months after transplantation into neonatal or adult rat hearts, whereas rat neonatal cardiomyocytes transplanted into rat hosts displayed more mature phenotypes, suggesting that matching graft and host species is important [113]. Indeed, in infarcted hearts of adult macaque monkeys, hiPSC-CMs developed an adult-like structure and contractile function after three months [20]. Conversely, Cho and colleagues showed that both mouse ESC-CMs and hiPSC-CMs transplanted into neonatal hearts of rats for one month showed mature morphology, T-tubule and intercalated disc-like structure formation, and faster calcium dynamics [114]. Therefore, more studies are needed to clarify these discrepancies. These studies demonstrate that hiPSC-CMs have the potential to achieve adult maturation. However, obvious graft-associated arrhythmias in non-human primates limited the therapeutic potential of hiPSC-CMs for repairing injured hearts [10, 19, 20]. Further disadvantages of directly applying this method to produce matured hiPSC-CMs include low cell viability after transplantation and the contamination of host cells, which makes them difficult to isolate.

### 3D culture

In recent years, 3D-culture systems such as engineered heart tissues (EHTs) and cardiac organoids have been developed to mimic cell–cell and cell–matrix interactions. Regardless of which approach was used to construct 3D cardiac tissues, hiPSC-CMs displayed structural, functional, physiological, and metabolic maturation compared to their 2D counterparts [115–118]. However, the use of 3D-culture systems per se is insufficient to achieve complete CM maturation. One of the potential issues is that hiPSC-CMs alone cannot create an appropriate extracellular matrix. To overcome this hurdle, Shadrin and colleagues generated a cardiac patch which included hiPSC-CM, SMCs, and CFs [118]. After 3 weeks in culture, hiPSC-CMs were characterized by organized structure, enhanced conduction velocity and contractile force, and the formation of T-tubule and intercalated disc-like structures [118]. Giacomelli and colleagues further combined hiPSC-CMs with hiPSC-CFs and -ECs to generate 3D micro-tissues. Their results showed that the addition of CFs and ECs significantly promoted hiPSC-CM maturation in terms of structure, electrophysiology, and metabolism. This was partly attributed to the tri-cellular crosstalk mediated by endothelin-1 and nitric oxide which were secreted from ECs and strengthened the coupling between hiPSC-CMs and CFs via connexin-43 gap junctions [119]. By using proteomic analysis and single-cell RNA sequencing, several recent studies have proposed that more complex intercellular signalling pathways, such as BMP4—BMPR1A, NRG3—ERRB4, and GDF11 (growth differentiation factor 11)—TGFRB1 (the former is the ligand from ECs and the latter is the receptor in hiPSC-CMs), between hiPSC-CMs and vascular cells are involved in promoting maturation [120, 121]. However, functional studies to confirm these results are currently lacking.

In the adult heart, the heartbeat is controlled by pacemaker cells in the sinoatrial node, whereas isolated healthy adult CMs do not beat spontaneously without electrical stimulation. In contrast, hiPSC-CMs show high automaticity and immature electrophysiological properties [122]. Several studies have revealed that external electrical stimulation yielded hiPSC-CMs with rod-like morphology and enhanced calcium handling [123, 124]. Interestingly, applying electric stimulation during cardiac differentiation not only enhanced the differentiation efficiency of hiPSC-CMs but also promoted their maturation [125, 126]. In terms of the combination with 3D culture, Nunes and colleagues developed 3D, self-assembled cardiac bio-wires by seeding hiPSC-CMs with fibroblasts, ECs, and SMCs into a sterile surgical suture and assessed hiPSC-CM maturation under two different electrical stimulation strategies: (1) a low-frequency ramp-up regimen, where stimulation started at 1 Hz, increased to 3 Hz (1 Hz, 1.83 Hz, 2.66 Hz and 3 Hz on days 1–4, respectively) and was maintained at 3 Hz for the remainder of the week, or (2) a high-frequency ramp-up regimen, where stimulation started at 1 Hz and increased to 6 Hz throughout the week (1 Hz, 1.83 Hz, 2.66 Hz, 3.49 Hz, 4.82 Hz, 5.15 Hz and 6 Hz) [127]. The results showed that the gradual increase from 1 to 6 Hz was the best stimulation condition in terms of enhancing hiPSC-CM maturation, as indicated by higher cardiac gene expression, organized sarcomeres, and improved calcium handling properties. However, their maturity was still not comparable with adult CMs, indicated by the absence of M-lines and T tubules [127].

Although electric stimulation can promote hiPSC-CM maturation, the limitation of this technique is that it can generate toxic Faradaic reactions at higher voltages and the in vitro apparatus is costly [128, 129]. In the last decade, the development of optogenetics has provided an alternative strategy to stimulate hiPSC-CMs. Optogenetic actuators can transform photon flux into transmembrane ion flux, thereby manipulating transmembrane potential on a millisecond scale [130, 131]. Quach and colleagues then generated hiPSC-CMs with the expression of ArchT (archaerhodopsin T, a hyperpolarizing opsin). By injecting an inward rectifier potassium current, hiPSC-CMs displayed a more negative resting membrane potential [132]. Another study employed a similar strategy to chronically optically paced EHTs for one week and reported improved electrophysiological properties in EHTs [133]. Lemme and colleagues further increased the pacing duration over 3 weeks and found that the paced EHTs had faster contraction kinetics, shorter action potentials, and shorter effective refractory periods compared with the control group [134]. Moreover, the improved electrophysiological properties of EHTs also lead to a high vulnerability to burst pacing-induced tachycardia, suggesting that matured hiPSC-CMs are more susceptible to pathogenic stimulation [134]. Overall, optogenetic tools show great potential to accelerate the development of more mature and physiologically relevant hiPSC-CMs.

In addition to stimuli from pacemaker cells, CMs are also exposed to increasing mechanical stress during development [135]. Dou and colleagues employed a micro-device array that can exert dynamic mechanical stimulation on hiPSC-CMs and quantitatively monitor cell contractility, and observed increased contractility and enhanced sarcomere structure under strains ranging from 1.54 to 14.79 kPa [136]. Further applying this mechanical loading to EHTs caused CM hypertrophy and alignment [137]. Recently, Ruan and colleagues combined electrical and mechanical strain in EHTs and found that molecular, structural, and force-generating properties displayed additive positive effects on maturation [138]. Ronaldson-Bouchard et al. invented a program called “intensity training” (two weeks at stimulation frequencies increasing from 2 to 6 Hz, followed by one week at 2 Hz) in combination with mechanical forces to mature EHTs [139]. The results showed that these hiPSC-CMs developed an adult-like cell size, T-tubules, and intermyofibrillar mitochondria with densely packed cristae [139]. Electrophysiological investigations showed a slightly less immature resting membrane potential, action potential, and upstroke velocity compared to adult CMs [139]. After that, a growing body of research using similar methods has shown that the combination of biophysical stimuli with 3D culture enabled the generation of adult-like heart tissues [140, 141]. Taken together, these studies confirmed that biophysical stimulation (mechanical or electric stimulation) applied to the 3D micro-tissues can further improve hiPSC-CM maturation.

Most of the 3D culture systems described above are EHTs or spheroids. In recent years, self-organizing cardiac organoids have drawn intensive attention as they recapitulate human cardiogenesis [142–145]. Although most studies were mainly focused on the developmental trajectory and downstream applications in disease modeling, several of them observed improved maturation of hiPSC-CMs in these self-organizing organoids [144, 146]. For instance, Silva and colleagues found that the presence of endoderm tissue (gut/intestine) in the hiPSC-derived organoids contributed to the functional maturation of hiPSC-CMs, indicated by larger cell size, elongated morphology, enhanced calcium handling, action potential amplitudes, and upstroke velocity, highlighting the importance of multi-tissue interactions for the physiological maturation of hiPSC-CMs [146]. In addition, Meier and colleagues recently utilized retinoic acid, a metabolite implicated in epicardial development, to promote the formation of epicardial cells and their derivatives to generate cardiac organoids showing self-organization of highly functional ventricular myocardium and epicardium [147]. Transcriptome analysis showed upregulated expression of the mature ventricular marker MYL2 by Day 15, a higher ratio of TNNI3/TNNI1, and increased expression of calcium-handling genes at Day 30, indicating progressive maturation [147]. Current challenges associated with studying cardiac organoids include difficulties in measuring the function of hiPSC-CMs in whole intact organoids due to their large size, high cell density and cellular heterogeneity. Furthermore, scaling up the generation of cardiac organoids in a reproducible way represents another challenge.

Collectively, these studies demonstrate that hiPSC-CMs are highly sensitive to the 3D environment as well as to biophysical cues. However, a notable limitation is that generating these engineered platforms is expensive, time-consuming, and challenging compared with traditional 2D monolayer culture.

## Conclusion and perspectives

**Fig. 2:**
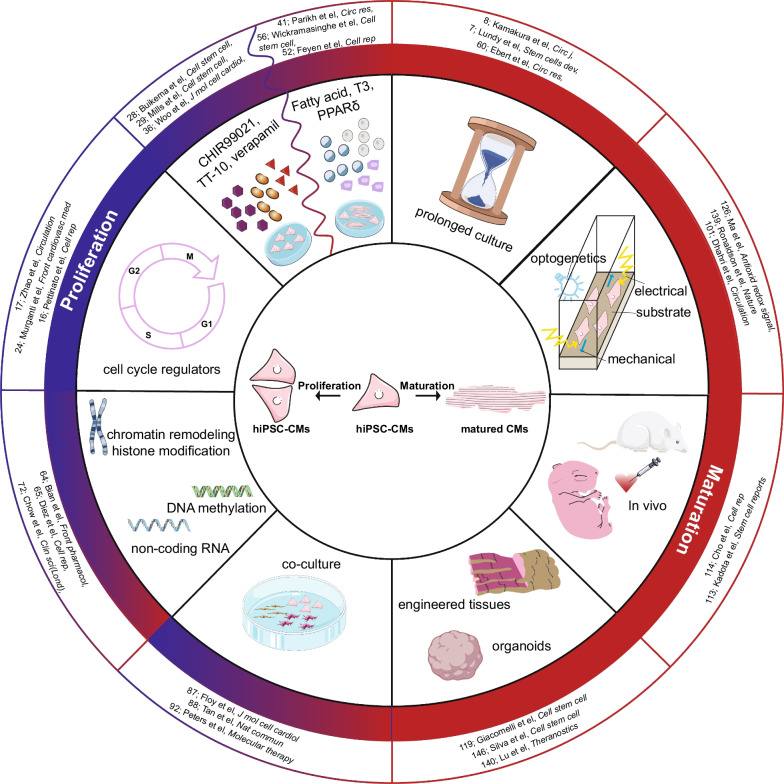
Overview of strategies that promote hiPSC-CM proliferation or maturation. The blue half circle indicates methods to promote hiPSC-CM proliferation. The red half circle indicates methods to promote hiPSC-CM maturation. The red-blue gradient indicates methods that provide pipelines to promote hiPSC-CM maturation or proliferation. The figure was drawn by the authors in Adobe Illustrator

Here, we reviewed multiple strategies that have been used to enhance the proliferation or maturation of hiPSC-CMs (Fig. 2). Since immature hiPSC-CMs are proliferative but matured hiPSC-CMs lack this capacity, directly scaling up the production of matured hiPSC-CMs is difficult. Therefore, sustaining or enhancing the cell cycle of hiPSC-CMs at an early stage is vital for promoting proliferation. Gene editing in cell cycle-related genes currently shows promising results in cell cycle maintenance and downstream application in cell therapy, but it is impossible to generate such autologous gene-edited hiPSC for human individuals. Moreover, special caution should be taken in using these proliferating hiPSC-CMs in drug screening and disease modeling, as they are probably immature and further studies should investigate whether this permanent cell cycle gene editing could hinder later stimulation of maturation or have undesired side-effects. Compared to the gene editing strategy, drug-induced proliferation might be a more appropriate method, as it is easy and controllable. The effect of drugs will reduce during long-term culture and treated hiPSC-CMs still retain the potential to be matured, which enables hiPSC-CM proliferation to be stimulated at an early stage before inducing their maturation and subsequent use. For other strategies of increasing proliferation such as epigenetic modifications, future studies should be focused on elucidating the underlying mechanisms, which may facilitate the discovery of pro-proliferative targets.

Many different methods have been developed to promote hiPSC-CM maturation in terms of structure, metabolism, and electrophysiology, and since all of these are related to mitochondrial function, this suggests that mitochondria play a key role in regulating CM maturation. Improved mitochondrial function has been observed in hiPSC-CMs following stimulation of maturation, but the mitochondria in these cells are not fully developed and incomparable to those of adult CMs [51, 148, 149], which might hinder their potential to acquire an adult-like phenotype. The development of methods involving intercellular mitochondrial transfer provides the possibility that transferring mitochondria from adult CMs into hiPSC-CMs might promote the generation of adult-like hiPSC-CMs [150–152]. Additionally, individual intervention strategies only promote one or several aspects of maturation, suggesting that maturation is driven by the coordinated regulation of multiple factors (Table 1). Moreover, chronological maturation of different cardiomyocyte structures and functions suggests that a mere combination approach may not be sufficient to formulate an effective maturation cocktail [153]. Further optimization of the timing, intensity, and duration of stimulating the maturation of individual cardiomyocyte properties might facilitate the generation of adult-like hiPSC-CMs, as 3D culture systems with biophysical stimulation have shown promise in terms of promoting maturation. Since the limitations of current engineered platforms are complex and costly, developing a reproducible, scalable, and cost-effective system remains a priority for achieving a high-throughput strategy.

**Table 1:** Summary of the effects of different methods on hiPSC-CM maturation and proliferation

Methods	Proliferation/Maturation	Effects	References
Cell cycle	Proliferation	Increase the number of Ki67, BrdU, pH3 and Aurora positive cells	[17, 18]
Small molecules	Proliferation	CHIR99021	[28, 29]
		Increased the number of Ki67 and pH3 positive cells	
		Decreased contractile force	
		Hit compounds from Hudson’s paper	
		Increased the number of Ki67 and pH3 positive cells	
Small molecules	Maturation	Fatty acid-based medium	[41, 51, 52]
		Increased contractile force	
		Enhanced oxidative and glycolytic metabolism	
		Enhanced calcium cycling	
		Highly negative resting membrane potential and increased AP upstroke velocity	
		T3 and glucocorticoids	
		Larger cell size	
		Improved myofibril organization	
		T-tubule formation	
		Increased contractile force	
		Enhanced calcium handling	
Prolonged culture	Maturation	Increased cell size	[7, 8]
		Increased number of MLC2v-positive/MLC2a-negative hiPSC-CMs	
		No detection of T-tubules	
Epigenetic regulation	Proliferation	miRNA	[64, 66, 83]
		Increased the number of Ki67, BrdU, pH3 and Aurora positive cells	
		m6A modification	
		Increased the number of EdU and pH3 positive cells	
Epigenetic regulation	Maturation	miRNA	[68, 72]
		Larger cell size	
		Increased contractile force	
		Enhanced oxidative metabolism	
		Histone modification	
		Larger cell size	
		Increased expression of genes related to ion channels	
Co-culture	Proliferation	Increased the number of Ki67 positive cells	[87, 88]
		Decreased sarcomere structural organization	
Co-culture	Maturation	Larger cell size	[93]
		Increased expression of genes related to ion channels	
Substrate stiffness	Maturation	Improved myofibril organization	[97, 98, 101]
		Increased contractile force	
		Enhanced oxidative metabolism	
		Enhanced calcium handling	
Physiological pattern	Maturation	Improved myofibril organization	[96, 105]
		T-tubule formation	
		Increased contractile force	
Conductivity	Maturation	Increased expression of genes related to ion channels	[108, 110]
		Enhanced calcium handling	
In vivo transplantation	Maturation	Larger cell size	[113, 114]
		Improved myofibril organization	
		T-tubule formation	
		Intercalated disc-like structure formation	
		Increased contractile force	
		Enhanced calcium handling	
Electric stimulation	Maturation	Larger cell size	[125, 127]
		Improved myofibril organization	
		Enhanced calcium handling	
Mechanical stress	Maturation	Larger cell size	[138, 139]
		Improved myofibril organization	
		Increased contractile force	
Optogenetics	Maturation	Enhanced calcium handling	[133, 134]
		Improved myofibril organization	
3D culture	Maturation	EHTs and spheroids	[118, 119, 146, 147]
		Larger cell size	
		Improved myofibril organization	
		T-tubule formation	
		Intercalated disc-like structure formation	
		Increased contractile force	
		Enhanced calcium handling	
		Enhanced oxidative metabolism	
		Organoids	
		Larger cell size	
		Enhanced calcium handling	
		Increased action potential amplitudes and upstroke velocity	

Although current matured hiPSC-CMs are still incomparable to adult CMs, they have indeed advanced the field of disease modeling, drug screening, and cell therapy. The fetal characteristics of hiPSC-CMs allow them to tolerate hypoxic conditions, whereas metabolically matured hiPSC-CMs show reduced mitochondrial respiration after exposure to hypoxia and increased cell death after increased duration of hypoxia, which may provide a good model for studying ischemia/reperfusion injury [154]. As another example, the use of conventional monolayer cultured hiPSC-CMs makes it harder to capture more clinically relevant phenotypes, especially for restrictive cardiomyopathy (RCM), which is characterized by impaired cardiac relaxation during diastole [155], a parameter which is difficult to measure in cells attached to a plastic substrate. By using EHTs, Wang and colleagues confirmed that a mutation in filamin C caused RCM phenotypes indicated by sarcomere disorganization, decreased active contraction force, and increased passive contraction force [156]. Subsequent drug screening identified that trequinsin, a phosphodiesterase 3 inhibitor, might be a potential drug which could ameliorate RCM phenotypes [156]. In terms of cell therapy, mature CMs have so far displayed poor engraftment within the host myocardium and low survival rate after transplantation, while immature hiPSC-CMs can cause severe engraftment arrhythmias. Therefore, choosing an optimal state of hiPSC-CMs with appropriate maturity and proliferative capacity is crucial for cell therapy. To achieve this aim, drug screening using mature hiPSC-CMs (such as EHTs and organoids) may help to discover drugs which can promote proliferative capacity without compromising cardiac function. Additionally, revascularization is important for the engraftment to survive in the host tissue. Several studies have reported that transplanted micro-vessels or endothelial cells significantly improved hiPSC-CM survival and their maturation compared to hiPSC-CMs transplanted alone in infarcted hearts, which was attributed to an improvement in revascularization and remuscularization [157, 158]. These findings suggest that transplanting vascularized cardiac tissues or organoids might have stronger effects on cardiac repair compared to the transplantation of a mixture of different cell types. However, this goal is hindered by the challenge of creating successful vascularized cardiac tissues or organoids. To address this issue, microfluidic devices could be used to enhance the vascular microenvironment, which has been demonstrated by successful vascularization of kidney and liver organoids [159, 160]. It is also important to note that several studies reported that transplanting hiPSC-CM derivatives is also beneficial for cardiac repair. Paracrine factors, mitochondrial transfer, and injection of exosomes were shown to improve cardiac remodeling by inhibiting apoptosis, regulating inflammation, and promoting angiogenesis [161–166]. Determining whether these derivatives from mature hiPSC-CMs could improve their function is valuable, as functional hiPSC-CM features such as mitochondria are underdeveloped in immature hiPSC-CMs. Moreover, whether co-transplantation of hiPSC-CMs and their derivatives can further promote cardiac repair warrants further investigation. Collectively, since different downstream applications require specific degrees of hiPSC-CM maturation, a more comprehensive investigation of the in vivo maturation process may help to generate optimal mature hiPSC-CMs for individual applications.

Overall, here we provide an overview of current research in hiPSC-CM proliferation and maturation, highlighting the knowledge gap and technical challenges that need to be addressed in the future.

## Data Availability

Data sharing is not applicable to this article as no datasets were generated or analyzed during the current study.

## Abbreviations

ABRO1: Abraxas 2
ALKBH5: AlkB homolog 5, RNA demethylase
BMPR: Bone morphogenetic protein receptor
CDKs: Cyclin-dependent kinases
CDK1: Cyclin-dependent kinase 1
CDK4: Cyclin-dependent kinase 4
CCNA2: Cyclin A2
CCNB1: Cyclin B1
CCNG1: Cyclin G1
CCND2: Cyclin D2
CFs: Cardiac fibroblasts
CFSE: Cytoplasmic carboxyfluorescein succinimidyl ester
CHIR: CHIR99021
CMM: Cardiac mimetic matrix
CPCs: Cardiac progenitor cells
ECG: Electrocardiogram
ECs: Endothelial cells
EdU: Thymidine 5-ethynyl-20-deoxyuridine
EHTs: Engineered heart tissues
EPIs: Epicardial cells
ERRγ: Estrogen-related receptor gamma
FSTL1: Follistatin like 1
H3K4me3: Trimethylation of lysine 4 on histone H3
hESC: Human embryonic stem cell
hESC-CMs: Human embryonic stem cell-derived cardiomyocytes
HIF1α: Hypoxia inducible factor 1 subunit alpha
hiPSCs: Human-induced pluripotent stem cells
hiPSC-CM: Human-induced pluripotent stem cell-derived cardiomyocyte
HSP90: Heat shock protein 90
IGF1: Insulin-like growth factor 1
KCNJ2: Potassium inwardly rectifying channel subfamily J member 2
LATS: Large tumor suppressor homolog
m6A: RNA N6-methyladenosine
METTL3: Methyltransferase 3, N6-adenosine-methyltransferase complex catalytic subunit
miRNAs: MicroRNAs
MST: Mammalian ste20-like kinases
MYL2: Myosin light chain 2
NRG1: Neuregulin 1β
PDMS: Polydimethylsiloxane
PG: Prostaglandin
PPAR: Peroxisome proliferator activated receptor
PKA: Protein kinase A
RCM: Restrictive cardiomyopathy
SCN5A: Sodium voltage-gated channel alpha subunit 5
SMCs: Smooth muscle cells
TGF-βR: Transforming growth factor β receptor
THs: Thyroid hormones
T3: Triiodothyronine
TNNT2: Troponin T2
TNNI3: Troponin I3
RYR2: Ryanodine receptor 2
